# Evolution of thyroid fine-needle aspiration biopsy performance over 15 years: implications for risk stratification in clinical practice

**DOI:** 10.20945/2359-4292-2026-0093

**Published:** 2026-08-12

**Authors:** Hakan Doğruel, Elif Nur Karaoğlu, Nusret Yılmaz, Mustafa Aydemir, Hasan Çalış, Cumhur Arıcı, Güzide Ayşe Ocak, İnanç Elif Gürer, Ramazan Sarı

**Affiliations:** 1 Division of Endocrinology and Metabolism, Department of Internal Medicine, Akdeniz University Faculty of Medicine, Antalya, Turkey; 2 Department of General Surgery, Akdeniz University Faculty of Medicine, Antalya, Turkey; 3 Department of Pathology, Akdeniz University Faculty of Medicine, Antalya, Turkey

**Keywords:** Biopsy, fine-needle, thyroid nodule, Bethesda system, diagnostic accuracy, cytopathology

## Abstract

**Objective:**

We aimed to evaluate temporal changes in the diagnostic performance of thyroid fine-needle aspiration biopsy (FNAB) over a 15-year period in a single-center, surgically confirmed cohort.

**Subjects and methods:**

We retrospectively analyzed thyroid FNABs performed between 2009 and 2023, dividing the data into two intervals (Group 1: 2009–2014; Group 2: 2015–2023). All specimens were interpreted by the same two experienced cytopathologists. Sensitivity, specificity, and Youden’s J were calculated at four diagnostic thresholds (≥AUS/FLUS, ≥FN/SFN, ≥SM, and malignant only), and level-specific likelihood ratios (LRs) were compared between cohorts. Overall diagnostic discrimination was assessed with receiver operating characteristic (ROC) curves and compared by DeLong’s test.

**Results:**

The study included 1,241 nodules; after excluding non-diagnostic cytology, 601 nodules in Group 1 and 553 in Group 2 were available for diagnostic performance analysis. At the ≥AUS/FLUS cutoff, sensitivity increased from 85.6% to 94.8%, while specificity declined from 58.4% to 33.0% (both p<0.01). Specificity at higher thresholds remained similar or increased in Group 2. The area under the ROC curve (AUC) was 0.799 for Group 1 and 0.831 for Group 2 (p = 0.24). Of the five Bethesda LRs, only that for AUS/FLUS changed significantly (1.24 vs. 0.37; p<0.01).

**Conclusion:**

The overall discriminative ability of the Bethesda System remained stable over 15 years. The decrease in the AUS/FLUS LR reflects a spectrum effect driven by more selective surgical referral patterns and should be interpreted as a change in cohort composition rather than diminished test performance.

## INTRODUCTION

Thyroid nodules are among the most frequently encountered endocrine pathologies, and their prevalence is rising with the increased use of imaging modalities (^1^,^2^). Fine-needle aspiration biopsy (FNAB) remains the cornerstone of initial risk stratification and management in patients with sonographically suspicious thyroid nodules (^1^,^3^). The Bethesda System for Reporting Thyroid Cytopathology (TBSRTC) provides a standardized framework for interpreting FNAB results and estimating the risk of malignancy within specific diagnostic categories (^4^,^5^)

However, discrepancies between cytological findings and final histopathological diagnoses persist, mainly due to sampling errors and interpretive challenges (^1^,^6^). These issues underscore the importance of institutional audits to assess the diagnostic accuracy and clinical utility of FNAB in real-world settings. Such audits are particularly valuable for evaluating temporal trends and the applicability of cytological classifications to local populations (^7^).

Previously, we conducted a study at our institution evaluating the concordance between FNAB cytology and final histopathology in patients who underwent thyroid surgery from 2009 to 2014, with additional analysis of the impact of nodule size on diagnostic accuracy (^6^). Since then, advances in biopsy techniques, ultrasonographic evaluation, and cytopathological reporting have been introduced (^8^,^9^). Therefore, in the present study, we aimed to evaluate the diagnostic performance of FNAB in patients who underwent thyroid surgery from 2015 to 2023, compare these results with our previously published data (2009–2014), and assess diagnostic performance using multiple cut-offs, receiver operating characteristic (ROC)-based discrimination, and prevalence-independent likelihood ratios (LRs).

## SUBJECTS AND METHODS

This retrospective study was conducted in the Department of Endocrinology and Metabolism. The study included all patients who underwent thyroid FNAB between January 1, 2015, and March 31, 2023, and subsequently underwent thyroid surgery with a final histopathological diagnosis available. The primary aim was to evaluate the diagnostic performance of FNAB and to compare the results with those from our previously published dataset covering the years 2009–2014, thereby assessing whether diagnostic performance changed over time with increasing institutional experience (^6^). All FNAB and surgical pathology specimens were consistently examined and reported by the same two pathologists throughout the study period. Patients who underwent FNAB and thyroid surgery between 2009 and 2014 were designated as Group 1, while those between 2015 and 2023 were assigned to Group 2. A comparative analysis of the data from both groups was subsequently performed to assess potential changes in diagnostic performance.

Patients were eligible for inclusion if they had undergone thyroid FNAB during the specified time period with available postoperative histopathological data. Patients who had incidental thyroid malignancy detected in non-biopsied nodules were excluded. Additionally, cases with non-diagnostic FNAB results were excluded from sensitivity, specificity, positive predictive value (PPV), negative predictive value (NPV), and accuracy analyses. Cases with missing data for specific variables were excluded from the corresponding analyses (variable-specific complete-case analysis).

For each patient, the following data were recorded: age, sex, thyroid function test results, thyroid autoantibody status, ultrasound-measured maximum nodule diameter, FNAB results, surgical indications, and final postoperative histopathological findings. FNAB results were classified according to the TBSRTC into six categories: nondiagnostic, benign, atypia of undetermined significance/follicular lesion of undetermined significance (AUS/FLUS), follicular neoplasm/suspicious for follicular neoplasm (FN/SFN), suspicious for malignancy (SM), and malignant. It should be noted that the AUS/FLUS terminology reflects the 2017 Bethesda System, which was in use during the study period; the 2023 revision, which eliminates the FLUS description, was introduced after completion of data collection (^4^,^5^). No molecular or immunocytochemical ancillary tests were performed in either time period, as such methods were not incorporated into routine practice at our institution during the study period.

In cases where multiple nodules were biopsied, each nodule was evaluated separately. If multiple FNABs were performed at different times for the same nodule, the highest-risk cytological category was used for classification. Postoperative specimens were classified as benign or malignant. For malignant cases, histological subtypes were recorded. Patients with incidental thyroid cancer in a nodule other than the one biopsied were excluded from the analysis.

### Statistical analysis

Statistical analyses were conducted using IBM SPSS Statistics version 23 (IBM Corp., USA), with additional calculations carried out in Python (version 3.11). Categorical variables are presented as frequencies and percentages, while continuous variables are presented as means with standard deviations. Group comparisons of categorical variables were performed using the chi-square or Fisher’s exact test, as appropriate. Continuous variables were compared using the independent-samples t-test.

To evaluate the diagnostic performance of FNAB, sensitivity, specificity, PPV, NPV, and overall accuracy were calculated using the histopathological reference standard. In the primary analysis, an FNAB result was considered positive if the cytological diagnosis was indeterminate (AUS/FLUS, FN/SFN, or SM) or malignant, and negative if benign; non-diagnostic specimens were excluded from the calculation of diagnostic indices. True positive (TP) cases were those with a positive FNAB and malignant final pathology; true negative (TN) cases were benign on both FNAB and final pathology; false positive (FP) cases had a positive FNAB but benign final pathology; and false negative (FN) cases had a benign FNAB but malignant final pathology. Sensitivity was calculated as TP / (TP + FN), specificity as TN / (TN + FP), PPV as TP / (TP + FP), NPV as TN / (TN + FN), and accuracy as (TP + TN) / (TP + TN + FP + FN). Wilson score 95% confidence intervals (CIs) were used for proportions (sensitivity, specificity, PPV, NPV, and accuracy). For LR, 95% CIs were derived from the normal approximation on the logarithmic scale, using the variance formula of Simel and cols. (^10^), a method recently adopted for standardizing biomarker interpretation in clinical cohorts (^11^). Diagnostic performance metrics for the 2015–2023 cohort (Group 2) were compared with those from the 2009–2014 cohort (Group 1) using two-proportion z-tests.

The analysis utilized four distinct cut-off values, as the choice of threshold for defining a positive FNAB result substantially influences the diagnostic performance of the test. The thresholds were AUS/FLUS or higher (Cut-off 1 (CO1), the conventional cut-off used in our primary analysis), FN/SFN or higher (CO2), suspicious for malignancy or higher (CO3), and malignant only (CO4). For each threshold and within each cohort, sensitivity, specificity, and Youden’s J index (calculated as sensitivity + specificity – 1) were derived, again with Wilson score 95% CIs, and the two cohorts were compared using two-proportion z-tests.

To assess the overall discriminative ability of the Bethesda System independent of any single cut-off, receiver operating characteristic (ROC) curve analysis was performed for each cohort, with the Bethesda category treated as an ordinal test variable and the final histopathological diagnosis as the reference standard. Areas under the curve (AUCs) and their nonparametric standard errors were obtained in SPSS (^12^). The two AUCs were compared using the method described by DeLong and cols., implemented in Python; the resulting test statistic and *p*-value were verified against a two-sample z-test using the SPSS-derived nonparametric variance estimates (^13^).

Because the prevalence of malignancy differed substantially between the two cohorts, predictive values were not directly comparable across periods; therefore, prevalence-independent measures were necessary to assess changes in test characteristics. We calculated level-specific LRs for each Bethesda category in both cohorts as LR = (a/M)/(b/N), where ‘a’ is the number of malignant cases in the category, ‘M’ is the total number of malignant cases, ‘b’ is the number of benign cases in the category, and ‘N’ is the total number of benign cases. The variance of the natural logarithm of LR was estimated using the formula proposed by Simel and cols. (^10^): Var(ln LR) = 1/a − 1/M + 1/b − 1/N . Between-cohort differences in category-specific LRs were tested using a z-test on the difference of ln(LR), with the standard error calculated as the square root of the sum of the two variances. Although the categories within a single cohort are not statistically independent, between-cohort comparisons of level-specific LRs are conventionally conducted using this approach. A two-sided *p*-value of less than 0.05 was considered statistically significant for all analyses.

In the size-stratified analysis, only sensitivity and specificity were compared between cohorts, as positive and negative predictive values are influenced by disease prevalence, which differed substantially between subgroups, and because subgroup sample sizes were insufficient to support stable, level-specific likelihood ratio estimation.

This study was prepared in accordance with the STROBE guidelines, and the completed checklist is provided in the Supplementary Material (^14^,^15^).

## RESULTS

The study included 1,241 nodules from patients who underwent thyroidectomy following FNAB, with 648 nodules in Group 1 and 593 in Group 2. The overall mean age was 49.7 ± 13.1 years, with a significant difference between Group 1 and Group 2 (48.2 ± 12.5 vs. 51.3 ± 13.6, *p* < 0.01). There was a female predominance overall (76.1%), with a modest but significant change in sex distribution between groups over time (*p* = 0.01). The proportion of nodules < 4 cm was significantly higher in Group 2 than in Group 1 (89.8% vs. 67.0%; *p* < 0.01). Similarly, the proportion of patients with positive thyroid autoantibodies was higher in Group 2 than in Group 1 (27.7% vs. 16.0%; *p* < 0.01). Normal thyroid-stimulating hormone (TSH) levels were more frequently observed in Group 2 (84.1% vs. 62.5%, *p* < 0.01) (**Table 1**).

**Table 1.** Baseline patient characteristics of the study cohort

Variable	Total (n = 1241)	Group 1 (n = 648)	Group 2 (n = 593)	*p*
Age (year) mean (±SD)	49.7 (±13.1)	48.2±12.5	51.3 (±13.6)	< 0.01
Sex, n (%)				
Female	944 (76.1)	511 (78.9)	433 (73.0)	0.01
Male	297 (23.9)	137 (21.1)	160 (27.0)	
Nodule diameter, n (%)				
<4 cm	964 (77.9)	434 (67.0)	530 (89.8)	< 0.01
≥ 4 cm	274 (22.1)	214 (33.0)	60 (10.2)	
Thyroid antibody, n (%)				
Negative	973 (78.4)	544 (84.0)	429 (72.3)	< 0.01
Positive	268 (21.6)	104 (16.0)	164 (27.7)	
Thyroid function, n (%)				
Normal TSH	904 (72.8)	405 (62.5)	499 (84.1)	< 0.01
Low TSH	215 (17.3)	164 (25.3)	51 (8.6)	
High TSH	122 (9.8)	79 (12.2)	43 (7.3)	

*p*-Values show the comparison between Groups 1 and 2. The largest nodule diameter data was missing for three nodules, all of which were in Group 2.

TSH: thyroid stimulating hormone.

**Figure 1.**
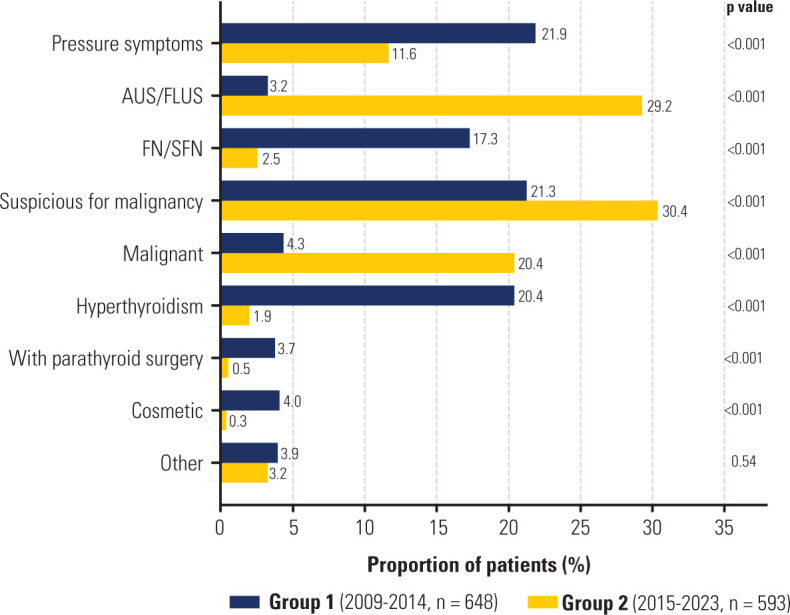
Indications for thyroidectomy in the study population by period. Bars represent the percentage of patients in each cohort for whom the listed indication was the primary reason for surgery. Group 1 (2009–2014) is shown in white with diagonal hatching, and Group 2 (2015–2023) in solid black. *p*-values represent two-proportion z-tests comparing the two periods. AUS/FLUS: Atypia of undetermined significance/follicular lesion of undetermined significance; FN/SFN: follicular neoplasm/suspicious for follicular neoplasm.

The most common overall indication for thyroidectomy was cytology SM (25.6%), followed by pressure symptoms (17.0%), AUS/FLUS (15.6%), and malignant FNAB (12.0%). These indications varied markedly between the two time periods. Notably, AUS/FLUS was a more frequent indication in Group 2 (29.2%) compared to Group 1 (3.2%, *p* < 0.01). Conversely, follicular neoplasm/suspicious for follicular neoplasm (FN/SFN) was a more common indication in Group 1 (17.3% vs. 2.5%, *p* < 0.01). Hyperthyroidism as an indication for thyroidectomy was also more prevalent in Group 1 than in Group 2 (20.4% vs. 1.9%, *p* < 0.01). Surgery for cosmetic reasons or during concomitant parathyroid procedures was rare (**Figure 1**).

Histopathological examination revealed benign lesions in 802 nodules (64.6%) and malignant lesions in 439 (35.4%). Papillary thyroid carcinoma was the predominant malignancy, accounting for 91.3% of malignant diagnoses. Other subtypes included medullary carcinoma (3.7%), follicular carcinoma (3.0%), anaplastic carcinoma (0.7%), and metastatic lesions (0.7%). The proportion of malignancy differed significantly between groups, rising from 22.1% in Group 1 to 49.9% in Group 2 (*p* < 0.01) (**Table 2**).

**Table 2.** Final histopathological diagnoses and distribution of malignant subtypes

Pathology	Total (n = 1241)	Group 1 (n = 648)	Group 2 (n = 593)	*p*
Benign, n (%)	802 (64.6)	505 (77.9)	297 (50.1)	< 0.01
Malignant, n (%)	439 (35.4)	143 (22.1)	296 (49.9)	< 0.01
PTC	401 (91.3)	126 (88.1)	275 (92.9)	0.01*
FTC	13 (3)	8 (5.6)	5(1.7)	
MTC	16 (3.6)	5 (3.5)	11 (3.7)	
Anaplastic	3 (0.7)	1 (0.7)	2 (0.7)	
Metastasis	3 (0.7)	3 (2.1)	0 (0.0)	
Unknown	3 (0.7)	-	3 (1.0)	

*p*-Values indicate the comparison between Groups 1 and 2. Histopathological subtype information for malignant nodules was unavailable for three patients in Group 2. Benign/malignant comparison: Pearson χ^2^ test.

PTC: papillary thyroid carcinoma; FTC: follicular thyroid carcinoma; MTC: medullary thyroid carcinoma.

* Fisher-Freeman-Halton exact test for subtype distribution (χ^2^ assumptions not met; 5/10 cells with expected count <5).

FNAB results were classified according to the Bethesda System. Malignancy rates by cytological category differed significantly between time periods. In the benign category, malignancy was observed less frequently in Group 1 than in Group 2 (6.5% vs. 14.7%, *p* = 0.01). For the FN/SFN category, the malignancy rate was lower in Group 1 than in Group 2 (15.8% vs. 42.9%, *p* = 0.03). The SM category showed a significantly lower malignancy rate in Group 1 than in Group 2 (44.9% vs. 71.2%, *p* < 0.01). No significant differences were observed in the AUS/FLUS, nondiagnostic, or malignant categories (**Table 3**).

**Table 3.** Bethesda category-specific malignancy rates across two time period

Variable	Total (n = 1241)		Group 1 (n = 648)		Group 2 (n = 593)		*p*
	FNAB result	MR	FNAB result	MR	FNAB result	MR	
Non-diagnostic, n (%)	87 (7.0)	18 (20.7)	47 (7.3)	11 (23.4)	40 (6.7)	7 (17.5)	0.68
Benign, n (%)	395 (31.8)	34 (8.6)	293 (45.2)	19 (6.5)	102 (17.2)	15 (14.7)	0.01
AUS/FLUS, n (%)	192 (15.5)	55 (28.6)	27 (4.2)	7 (25.9)	165 (27.8)	48 (29.1)	0.91
FN/SFN, n (%)	128 (10.3)	24 (18.8)	114 (17.6)	18 (15.8)	14 (2.4)	6 (42.9)	0.03
SM, n (%)	301 (24.3)	178 (59.1)	138 (21.3)	62 (44.9)	163 (27.5)	116 (71.2)	<0.01
Malign, n (%)	138 (11.1)	130 (94.2)	29 (4.5)	26 (89.7)	109 (18.4)	104 (95.4)	0.46

*p*-Values show the comparison of malignancy rates between Groups 1 and 2.

AUS: Atypia of undetermined significance; FLUS: follicular lesion of undetermined significance; MR: malignancy rate; FN: follicular neoplasm; SFN: suspicious for follicular neoplasm; SM: suspicious for malignancy.

Level-specific LRs were calculated for each Bethesda category. The LR for the AUS/FLUS category was 1.24 (95% CI, 0.54–2.88) in Group 1 and 0.37 (95% CI, 0.28–0.50) in Group 2 (Z = 2.65, *p* < 0.01). For other categories, LRs were 0.25 in Group 1 and 0.16 in Group 2 for benign (*p* = 0.19), 0.67 and 0.69 for FN/SFN (*p* = 0.96), 2.90 and 2.25 for SM (*p* = 0.22), and 30.79 and 19.00 for malignant (*p* = 0.52) (**Table 4**).

**Table 4.** Level-specific likelihood ratios for each Bethesda category, by cohort

Bethesda category	Group 1 (n = 601)		Group 2 (n = 553)		Z	*p*
	LR	95% CI	LR	95% CI		
Benign	0.25	0.16–0.38	0.16	0.09–0.27	+1.31	0.192
AUS/FLUS	1.24	0.54–2.88	0.37	0.28–0.50	+2.65	<0.01
FN/SFN	0.67	0.42–1.06	0.69	0.24–1.95	-0.05	0.96
SM	2.90	2.20–3.81	2.25	1.68–3.03	+1.22	0.22
Malignant	30.79	9.47–100.15	19.00	7.87–45.89	+0.64	0.52

Likelihood ratios were calculated from the category-specific cancer and non-cancer counts in **Table 3**, using the formula LR = (a/M)/(b/N), where a is the number of malignant cases in the category, M is the total number of malignant cases, b is the number of benign cases in the category, and N is the total number of benign cases. The variance of ln(LR) was estimated as 1/a − 1/M + 1/b − 1/N. Between-cohort comparisons were conducted using a z-test on the difference of ln(LR).

LR: Likelihood ratio; CI: confidence interval; AUS/FLUS: atypia of undetermined significance/follicular lesion of undetermined significance; FN/SFN: follicular neoplasm/suspicious for follicular neoplasm; SM: suspicious for malignancy.

When FNAB diagnostic performance was assessed using the conventional threshold of AUS/FLUS or higher as a positive result (CO1), sensitivity increased significantly from Group 1 to Group 2 (85.6% vs. 94.8%; *p* < 0.01), whereas specificity declined (58.4% vs. 33.0%; *p* < 0.01). At CO2, sensitivity was 80.3% in Group 1 and 78.2% in Group 2 (*p* = 0.62), and specificity was 62.7% and 77.3%, respectively (*p* < 0.01). At CO3, sensitivity was 66.7% in Group 1 and 76.1% in Group 2 (*p* = 0.04), while specificity was 83.2% and 80.3% (*p* = 0.33). At CO4, sensitivity was 19.7% in Group 1 and 36.0% in Group 2 (*p* < 0.01), with specificity of 99.4% and 98.1% (*p* = 0.12). Youden’s J index was 0.440 in Group 1 and 0.278 in Group 2 at CO1; 0.430 and 0.555 at CO2; 0.498 and 0.564 at CO3; and 0.191 and 0.341 at CO4 (**Table 5**).

**Table 5.** Multi-cutoff diagnostic performance of thyroid FNAB across two periods

CO	Group	Sensitivity, % (95% CI)	Specificity, % (95% CI)	Youden’s J	PPV, % (95% CI)	NPV, % (95% CI)	Accuracy, %
CO1	1	85.6 (78.6–90.6)	58.4 (53.9–62.8)	0.440	36.7 (31.5–42.2)	93.5 (90.1–95.8)	64.4
	2	94.8 (91.6–96.8)	33.0 (27.6–38.8)	0.278	60.8 (56.2–65.2)	85.3 (77.1–90.9)	65.3
	*p-value*	<0.01	<0.01	—	<0.01	0.01	0.75
CO2	1	80.3 (72.7–86.2)	62.7 (58.2–66.9)	0.430	37.7 (32.3–43.5)	91.9 (88.4–94.4)	66.6
	2	78.2 (73.1–82.6)	77.3 (71.8–81.9)	0.555	79.0 (73.9–83.3)	76.4 (71.0–81.1)	77.8
	*p-value*	0.62	<0.01	—	<0.01	<0.01	<0.01
CO3	1	66.7 (58.3–74.1)	83.2 (79.5–86.3)	0.498	52.7 (45.1–60.1)	89.9 (86.7–92.4)	79.5
	2	76.1 (70.9–80.7)	80.3 (75.1–84.7)	0.564	80.9 (75.8–85.1)	75.4 (70.1–80.1)	78.1
	*p-value*	0.04	0.33	—	<0.01	<0.01	0.55
CO4	1	19.7 (13.8–27.3)	99.4 (98.1–99.8)	0.191	89.7 (73.6–96.4)	81.5 (78.1–84.4)	81.9
	2	36.0 (30.7–41.7)	98.1 (95.6–99.2)	0.341	95.4 (89.7–98.0)	58.3 (53.7–62.8)	65.6
	*p-value*	<0.01	0.12	—	0.23	<0.01	<0.01

Diagnostic performance metrics were calculated after excluding nodules with non-diagnostic cytology. Cut-off definitions: CO1, Bethesda category III and above classified as positive; CO2, Bethesda category IV and above classified as positive; CO3, Bethesda category V and above classified as positive; CO4, Bethesda category VI classified as positive. Ninety-five percent confidence intervals were computed using the Wilson score method. *p*-values represent two-proportion z-tests comparing Group 1 with Group 2.

CO: Cut-off; PPV: positive predictive value; NPV: negative predictive value; CI: confidence interval; AUS: atypia of undetermined significance; FLUS: follicular lesion of undetermined significance; FN: follicular neoplasm; SFN: suspicious for follicular neoplasm; SM: suspicious for malignancy.

**Figure 2.**
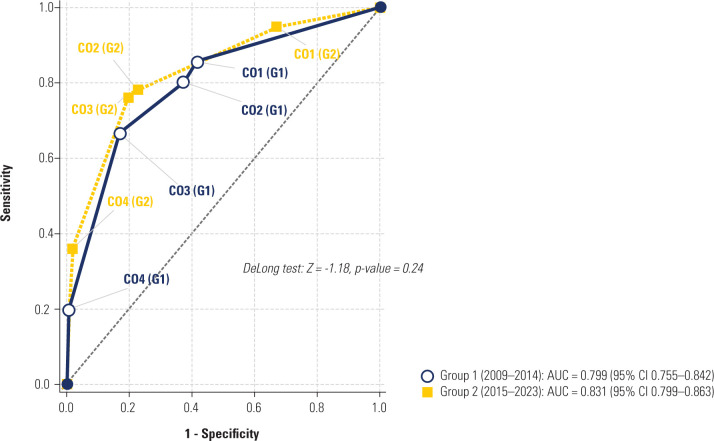
Receiver operating characteristic curves comparing the performance of thyroid fine-needle aspiration biopsy (FNAB) in Group 1 (2009–2014) and Group 2 (2015–2023). The Bethesda category was used as an ordinal test variable, with final histopathology serving as the reference standard. Each vertex along the curves corresponds to one of four cut-off thresholds for defining a positive FNAB result: CO1, ≥AUS/FLUS; CO2, ≥FN/SFN; CO3, ≥SM; CO4, malignant only. Areas under the curve (AUC) and 95% confidence intervals are provided in the legend. The diagonal dotted line represents an uninformative test. A comparison of the two AUCs using the DeLong test for two independent samples revealed no statistically significant difference (Z = −1.18,* p* = 0.238), indicating that the overall discriminative performance of the Bethesda System remained stable between the two periods.

ROC curve analysis was performed for each cohort using the Bethesda category as an ordinal test variable. The AUC was 0.799 (95% CI, 0.755–0.842) in Group 1 and 0.831 (95% CI, 0.799–0.863) in Group 2. The difference of 0.032 was not statistically significant (DeLong test, Z = −1.18, *p* = 0.24) (**Figure 2**).

Stratification by nodule size was performed using CO1, with comparisons limited to sensitivity and specificity. For nodules ≥4 cm, sensitivity was 75.9% in Group 1 and 81.8% in Group 2 (*p* = 0.60), while specificity was 75.2% and 56.2% (*p* = 0.02). For nodules <4 cm, sensitivity was 88.3% in Group 1 and 95.9% in Group 2 (*p* < 0.01), while specificity was 49.3% and 30.1% (*p* < 0.01). Youden’s J index was 0.510 in Group 1 and 0.381 in Group 2 for nodules ≥4 cm, and 0.377 and 0.260 for nodules <4 cm (**Table 6**).

**Table 6.** Temporal comparison of thyroid FNAB diagnostic performance by nodule size

Variable	Nodule size ≥4 cm			Nodule size <4 cm		
	Group 1 (n = 194)	Group 2 (n = 54)	*p*	Group 1 (n = 407)	Group 2 (n = 496)	*p*
Sensitivity* (95% CI)	75.9 (57.9–87.8)	81.8 (61.5–92.7)	0.60	88.3 (80.7–93.2)	95.9 (92.8–97.7)	<0.01
Specificity* (95% CI)	75.2 (68.0–81.1)	56.2 (39.3–71.8)	0.02	49.3 (43.8–54.9)	30.1 (24.6–36.4)	<0.01
Youden’s J	0.510	0.381	-	0.377	0.260	-

Diagnostic indices in this stratified analysis were calculated using the conventional ≥AUS/FLUS cut-off only, after excluding nodules with non-diagnostic cytology. Nodule diameter was missing for three patients in Group 2, who were therefore excluded from this stratified analysis. 95% Confidence intervals were computed using the Wilson score method. *p*-values represent two-proportion z-tests comparing Group 1 with Group 2 for sensitivity and specificity. Youden’s J index is provided as a descriptive summary of the sensitivity–specificity balance (calculated as sensitivity + specificity - 1) and is not subjected to formal statistical comparison between cohorts. Positive predictive value, negative predictive value, and accuracy were excluded from this stratified analysis because they are influenced by disease prevalence, which differed substantially between subgroups. Subgroup sample sizes, particularly the 54 cases of nodules ≥4 cm in Group 2, were insufficient to support stable level-specific likelihood ratio estimation or multi-cutoff ROC-based analysis. CI: confidence interval.

## DISCUSSION

In this large, 15-year retrospective cohort study, the diagnostic performance of thyroid FNAB exhibited a temporal shift in the sensitivity-specificity balance, rather than a uniform improvement in overall accuracy. At the conventional CO1 threshold, sensitivity increased from 85.6% to 94.8%, while specificity decreased from 58.4% to 33.0%. However, multi-cutoff analysis revealed that this loss of specificity was limited to the CO1 threshold; at higher cutoffs (CO2, CO3, or CO4), specificity in the recent cohort was comparable to, or higher than, that in the earlier period. This shift was also evident in the level-specific LR for the AUS/FLUS category, which declined from 1.24 to 0.37 (*p* < 0.01). Because likelihood ratios are mathematically independent of disease prevalence, this finding cannot be attributed to the increase in the overall malignancy rate from 22.1% to 49.9%. A spectrum effect is a more plausible explanation. After the 2015 American Thyroid Association (ATA) guidelines (^16^), FNAB and surgical referral practices shifted from a size-based approach to one based on sonographic risk stratification. Consequently, the case mix of benign and malignant nodules reaching surgery shifted toward higher-suspicion lesions. As a result, the category-specific conditional probabilities that define the LR changed, even though cytopathological interpretations continued to be performed by the same two pathologists throughout the study period.

The LR estimate for Group 1 was also statistically unstable; its 95% CI (0.54–2.88) crossed unity, indicating that the point estimate of 1.24 was not well established. The Group 2 CI (0.28–0.50) was considerably narrower, situating AUS/FLUS within the low-risk category for this surgical cohort. Clinically, in a cohort with an initial probability of malignancy of approximately 50%, an AUS/FLUS LR produces a post-test probability of 29.1%, below the cohort average. This demonstrates that the interpretation of an indeterminate cytological category depends on the pre-test probability of the population in which it is applied. The LRs reported in this study should, therefore, be considered as characteristics of this surgically confirmed cohort rather than as population-level estimates.

Moreover, the area under the ROC curve remained stable (0.799 vs. 0.831, *p* = 0.24), indicating that the overall discriminative ability of the Bethesda System did not change significantly. These findings suggest that the divergence in metrics observed at the conventional CO1 threshold reflects a shift in the operating characteristics for clinical decision-making rather than a global change in diagnostic power. The substantial increase in PPV from 36.7% to 60.8% should be interpreted with caution, as it was driven by a more than twofold increase in malignancy prevalence within this surgically confirmed cohort. This finding aligns with recent meta-analyses in high-resource settings (^17^) and underscores the importance of evaluating FNAB performance across multiple thresholds and in various local prevalence contexts. Importantly, the LRs reported here should be regarded as characterizing the present surgically confirmed population, not as updated population-level estimates for the Bethesda System as a whole.

The shift in cohort composition that underlies these findings was driven by significant changes in surgical indications over time. There was a decrease in surgeries for hyperthyroidism and benign nodular disease, coupled with an increase in procedures prompted by cytological suspicion, such as AUS/FLUS, SM, and malignant cytology (Figure 1). This likely reflects increasing reliance on FNAB-based decision-making and more selective surgical referral, has led to a more “malignancy-enriched” surgical cohort. This may account for the concurrent rise in the proportion of malignant nodules among resected specimens observed in this study and other recent reports (^18^–^20^).

Malignancy rates across Bethesda categories showed distinct temporal shifts. The most pronounced change was seen in the FN/SFN category, where the malignancy rate nearly tripled from 15.8% to 42.9%, exceeding the traditionally expected range (^21^–^24^). A similar trend was observed in the SM category, with an increase from 44.9% to 71.2%, consistent with existing literature (^1^,^5^,^22^). These increases are likely attributable to more selective surgical referral, rather than to fundamental changes in cytopathological interpretation. Supporting this, level-specific LRs for the benign, FN/SFN, SM, and malignant categories did not differ significantly between periods (Table 4), suggesting that the inherent discriminative value of these categories remained stable despite apparent shifts in malignancy rates. The AUS/FLUS malignancy rate remained stable between periods, though it exceeded the classically cited range in both cohorts (^1^,^5^). Notably, the 2016 reclassification of non-invasive encapsulated follicular variant of papillary thyroid carcinoma as noninvasive follicular thyroid neoplasm with papillary-like nuclear features may have affected historical malignancy estimates, particularly in the earlier cohort, where such lesions would have been classified as malignant (^25^).

The elevated malignancy rate in Bethesda II is also noteworthy. Both cohorts exceeded the 0–3% range reported in the 2017 TBSRTC (^5^); however, only surgically treated nodules were included in this study, altering the interpretation. In routine practice, Bethesda II nodules rarely undergo surgery; when they do, it is usually because of suspicious sonographic features, compressive symptoms, cosmetic concerns, or a coexisting nodule requiring surgery. Thus, the observed malignancy rate reflects a selection effect; only Bethesda II nodules with additional clinical or sonographic concerns are surgically excised. Two further factors may contribute to this finding. First, exact nodule-level matching in multinodular glands is challenging, so a small proportion of cases may represent incidental carcinoma in adjacent nodules. Second, tertiary referral centers see a higher pre-test probability of malignancy than community settings (^26^), thereby increasing malignancy rates in all cytological categories, including benign.

Several factors may underlie the differences observed between the two cohorts. Cytopathology was consistently performed by the same two pathologists throughout the study, limiting potential interobserver variability. Nevertheless, clinical practice evolved, with higher-resolution ultrasound becoming available in the later period. FNABs were performed, throughout, by endocrinology fellows rotating during their three-year training, so operator experience varied within—though not systematically between—the two cohorts. The decline in specificity was confined to CO1; at CO2, CO3, and CO4 thresholds, specificity was higher or unchanged. Together with the absence of a significant difference in AUC, these results suggest that the overall discriminatory power of the Bethesda System to distinguish malignant from benign nodules was preserved. Similarly, level-specific LRs were unchanged across four of the five Bethesda categories (Table 4). The exception, AUS/FLUS, is better explained by the shift in cohort composition than by a change in test characteristics.

Stratification by nodule size revealed a consistent pattern. In both subgroups, specificity declined and Youden’s J index decreased at the CO1 threshold, consistent with the unstratified analysis (Table 6), though the small number of nodules ≥4 cm in Group 2 (n = 54) limits the strength of any conclusions drawn from this subgroup.

Our study has several strengths. Conducted over 15 years at a single institution, all FNABs and surgical specimens were interpreted by the same two experienced cytopathologists; this design is uncommon in the longitudinal FNAB literature. Previous studies comparing pre- and post-Bethesda periods typically involved multiple observers or shorter follow-up intervals (^27^–^29^), complicating temporal comparison. The present study also combined three analytic approaches that are rarely used together in this context: multi-cutoff evaluation across four thresholds, ROC-based comparison of discriminative performance between periods, and prevalence-independent level-specific LRs. This comprehensive analysis offers a more nuanced perspective on changes in FNAB performance over time than single-threshold sensitivity–specificity analyses.

This study has several limitations. First, as a single-center retrospective analysis at a tertiary referral hospital, findings may not be generalizable to broader clinical practice. Second, only surgically treated nodules were included, resulting in higher malignancy rates than would be observed in an unselected biopsy population. Third, the 2016 reclassification of non-invasive follicular variant papillary thyroid carcinoma as noninvasive follicular thyroid neoplasm with papillary-like nuclear features could not be retrospectively applied to the earlier cohort, potentially inflating historical malignancy rates. Fourth, the sample sizes for size-stratified analysis and the FN/SFN category in Group 2 were small (n = 54 and n = 14, respectively), warranting cautious interpretation of those subgroups. Finally, no molecular or immunocytochemical ancillary testing was performed during the study period, limiting comparability with centers where such diagnostic modalities are available for indeterminate nodules.

In conclusion, this 15-year study demonstrates that the Bethesda System’s ability to distinguish malignant from benign thyroid nodules remained stable over time, as shown by multi-cutoff analyses, ROC curves, and level-specific LRs. The only exception, the decline in the AUS/FLUS LR, is most likely explained by a spectrum effect rather than a change in cytopathologic interpretation. This likely reflects a shift toward a more selective, sonographically risk-stratified surgical population following the introduction of the 2015 ATA guidelines. As malignancy rates in surgical series are shaped by local referral patterns, these LRs should be interpreted within that specific context and not generalized to unselected biopsy populations.

## Data Availability

the datasets used and/or analyzed during the current study are available from the corresponding author upon reasonable request.
